# PUMA facilitates EMI1-promoted cytoplasmic Rad51 ubiquitination and inhibits DNA repair in stem and progenitor cells

**DOI:** 10.1038/s41392-021-00510-w

**Published:** 2021-03-31

**Authors:** Jin Wook Kang, Zhiyan Zhan, Guangzhen Ji, Youzhou Sang, Daohong Zhou, Yanxin Li, Haizhong Feng, Tao Cheng

**Affiliations:** 1 State Key Laboratory of Experimental Hematology, Institute of Hematology & Blood Diseases Hospital, Center for Stem Cell Medicine, Chinese Academy of Medical Sciences and Peking Union Medical College, Tianjin, China; 2 Key Laboratory of Pediatric Hematology and Oncology Ministry of Health, Department of Hematology & Oncology, Shanghai Children’s Medical Center, Shanghai Jiao Tong University School of medicine, Shanghai, China; 3 Renji-Med X Clinical Stem Cell Research Center, Ren Ji Hospital, Shanghai Cancer Institute, School of Medicine, Shanghai Jiao Tong University, Shanghai, China; 4 Department of Pharmacodynamics, College of Pharmacy, University of Florida at Gainesville, Gainesville, FL USA

**Keywords:** Embryonic stem cells, Haematopoietic stem cells

## Abstract

Maintenance of genetic stability via proper DNA repair in stem and progenitor cells is essential for the tissue repair and regeneration, while preventing cell transformation after damage. Loss of *PUMA* dramatically increases the survival of mice after exposure to a lethal dose of ionizing radiation (IR), while without promoting tumorigenesis in the long-term survivors. This finding suggests that PUMA (p53 upregulated modulator of apoptosis) may have a function other than regulates apoptosis. Here, we identify a novel role of PUMA in regulation of DNA repair in embryonic or induced pluripotent stem cells (PSCs) and immortalized hematopoietic progenitor cells (HPCs) after IR. We found that *PUMA*-deficient PSCs and HPCs exhibited a significant higher double-strand break (DSB) DNA repair activity via Rad51-mediated homologous recombination (HR). This is because PUMA can be associated with early mitotic inhibitor 1 (EMI1) and Rad51 in the cytoplasm to facilitate EMI1-mediated cytoplasmic Rad51 ubiquitination and degradation, thereby inhibiting Rad51 nuclear translocation and HR DNA repair. Our data demonstrate that PUMA acts as a repressor for DSB DNA repair and thus offers a new rationale for therapeutic targeting of PUMA in regenerative cells in the context of DNA damage.

## Introduction

Stem cells are undifferentiated cells with the potential of self-renewal and differentiation into various kinds of cell types during the development and lifetime. Progenitor cells are intermediate proliferative cells that can further mature into functional cells in a specific lineage(s). Because stem and progenitor cells are responsible for the tissue regeneration under homeostatic conditions and after injury, respectively, any mis-repaired DNA damage in these cells can be transmitted to their differentiated progeny, thus compromising tissue integrity and function.^1^ Therefore, a proper DNA repair capacity is required for stem or progenitor cells to maintain the genomic stability.^2^ However, if these cells have enhanced ability to use error-prone DNA repair mechanism to repair DNA damage, it can lead to genetic instability to facilitate the formation of tumor-initiating cells or tumor stem cells.^3^

PUMA (p53 upregulated modulator of apoptosis) is a direct p53 target gene that encodes a BH3-only proapoptotic protein.^4–6^ It has been reported that lymphoid cells,^7^ myeloid hematopoietic progenitor cells (HPCs),^8^ and intestinal progenitor cells^9^ are resistant to ionizing radiation (IR) in the absence of PUMA. Moreover, inactivation of PUMA provides significant radioprotection at the level of hematopoietic stem cells,^10^ thereby conferring striking long-term survival of the exposed mice after lethal dose of IR. Interestingly and puzzlingly, no increase of hematopoietic malignancies was observed in the long-term survived animals after exposure to a high-dose of IR.^10^ These striking and unique phenotypes cannot be explained by the reduced apoptosis in the stem and progenitor cell compartments in the absence of PUMA. We hypothesize that, the no increased malignancy may be at least in part attributable to an enhanced DNA repair in the *PUMA*-deficient cells. This study was designed to test this hypothesis.

In the present study, we show that *PUMA* knockout (KO) pluripotent stem cells (PSCs) and HPCs underwent enhanced double-strand break (DSB) repair via homologous recombination (HR) and nonhomologous end joining (NHEJ) after exposure to IR. *PUMA* KO cells preferentially used HR repair because they express higher levels of nuclear Rad51. We show that ectopic expression of PUMA reduces Rad51 expression and enhances Rad51 ubiquitination and degradation, whereas depletion of *PUMA* results in a high level of Rad51 in association with a reduction in Rad51 ubiquitination. Moreover, PUMA-mediating Rad51 ubiquitination was dependent on EMI1 (early mitotic inhibitor 1; also known as FBXO5 and FBX5), an F-box protein,^11^ which has been shown to be involved in the regulation of Rad51 ubiquitination and HR DNA repair in breast cancer.^12^ We found that PUMA formed a ternary complex with Rad51 and EMI1 in the cytoplasm, and facilitated Rad51 ubiquitination by EMI1 in PSCs and HPCs. Taken together, our results demonstrate that PUMA can repress Rad51-mediated HR repair via promoting its ubiquitination by EMI1. Therefore, targeted inhibition of PUMA has dual beneficial effects against IR, i.e., protecting the cells from IR-induced apoptosis and maintaining genomic instability.

## Results

### *PUMA* deficiency increases DNA repair in both PSCs and HPCs following IR

**Fig. 1:**
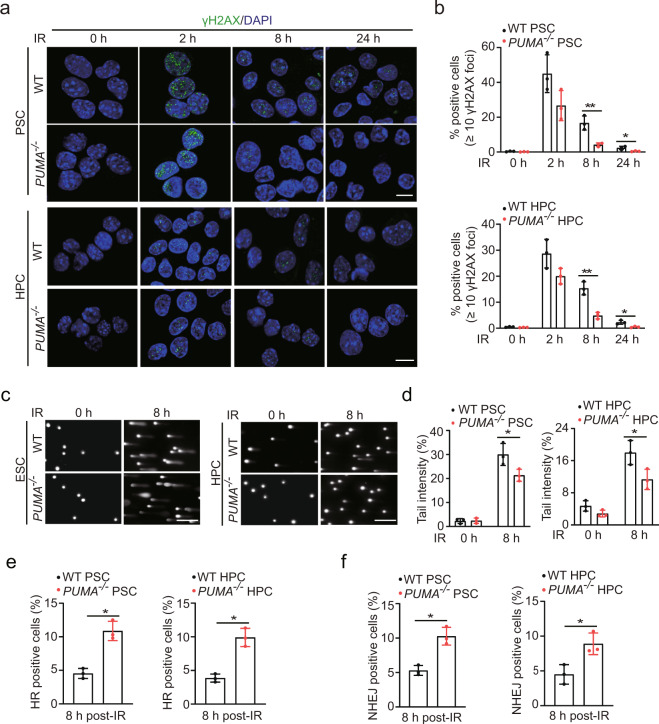
Knockout of *PUMA* enhances IR-induced DNA repair in PSCs and HPCs. **a** Representative images of γH2AX foci in *PUMA* wild type (WT) and KO PSCs and HPCs after IR. Scale bars, 10 μm. PSCs and HPCs were treated with 2 Gy IR and then fixed at 0, 2, 8, or 24 h for γH2AX staining. **b** Quantification of γH2AX foci in **a**. The percentage of positive cells (≥10 γH2AX foci) is shown. **c** Representative images of comet tails in *PUMA* WT and KO PSCs or HPCs at indicated time points after treated with 2 Gy of IR. Scale bars, 250 μm. **d** Quantification of comet tail intensity in **c**. **e**, **f** Quantification of HR (**e**) or NHEJ (**f**)-positive cells in *PUMA* WT and KO PSCs or HPCs. At 48 h after I-*Sce*I transfection, *PUMA* WT or KO PSC and HPC cells with stable expression of a pDR-GFP or pEJ5-GFP reporter were treated with 2 Gy IR and then harvested at 8 h for analysis, using flow cytometry to examine recombination induced by I-*Sce*I digestion. Dead cells were excluded by PI staining. Data are representative of three independent experiments with similar results. Error bars, SD. *P < 0.05, **P < 0.01

γH2AX foci have been widely used as a sensitive indicator for DNA DSB.^13^ We used immunofluorescence staining to quantify γH2AX foci-positive cells in response to IR. The number of γH2AX foci-positive cells in unirradiated *PUMA* WT and KO PSCs and HPCs was low, but increased immediately after 2 Gy IR (Fig. 1a, b). At 2 and 8 h post-IR, the percentage of γH2AX foci-positive cells significantly decreased in *PUMA* KO PSCs compared to that in WT PSCs (Fig. 1a, b). Flow cytometry analysis also showed that the percent of γH2AX-positive cells in *PUMA* KO PSCs following IR were lower than that in WT PSCs (Supplementary Fig. 1a, b). The persistence of γH2AX foci in WT cells after IR indicates that some of the damage remains unrepaired.^14^ Alkaline comet assay was performed and the results from this assay confirmed the finding with γH2AX foci assay (Fig. 1c, d). To rule out the compounding effect of less apoptosis in the absence of PUMA, we also quantified Annexin V-positive apoptotic cells at 8 h post-IR (2 Gy) and found that the number of apoptotic cells in WT PSCs were comparable to those in *PUMA* KO PSCs (Supplementary Fig. 1c, d). These results demonstrate that PUMA inhibits IR-induced DNA repair in PSCs and HPCs.

Two major pathways involved in the repair of DSBs in eukaryotic cells are HR and NHEJ. Accumulating evidence suggests that HR and NHEJ cooperate and compete with each other at DSB sites to facilitate efficient repair and promote genomic integrity.^15^ To investigate the mechanisms by which *PUMA KO* enhances DSB repair in PSCs and HPCs, we generated *PUMA* WT and *KO* PSCs and HPCs stably expressing HR reporter DR-GFP or NHEJ reporter EJ5-GFP, and then performed HR and NHEJ analysis. As shown in Fig. 1e, the percentages of HR-repaired cells in *PUMA KO* PSCs significantly increased compared to that in WT PSCs. A similar result for NHEJ repair was found in *PUMA* WT and KO HPC (Fig. 1f). These data demonstrate that PUMA mediates DSB repair via both HR and NHEJ in stem cells.

### *PUMA*-deficient PSCs and HPCs express elevated Rad51 compared to *PUMA* WT

**Fig. 2:**
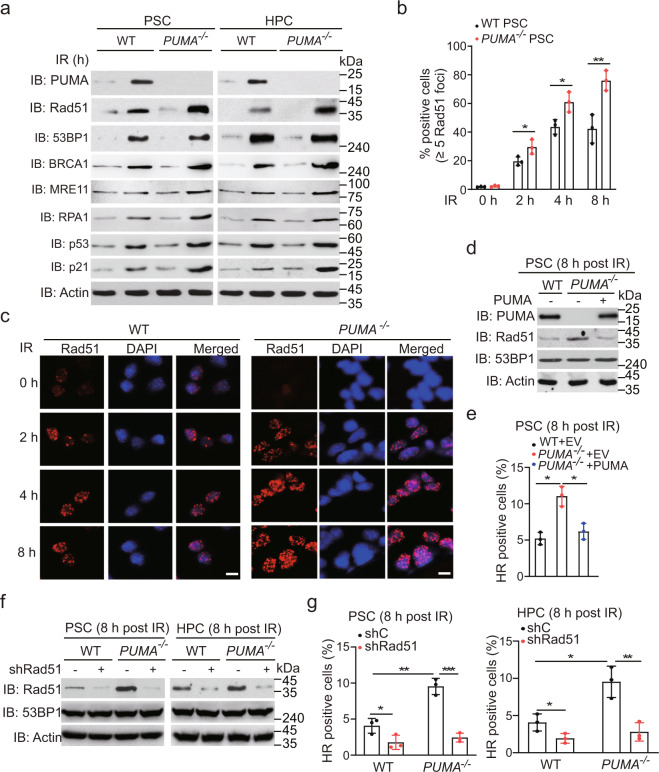
Knockout of *PUMA* elevates Rad51 in PSCs or HPCs. **a** Immunoblotting (IB) of Rad51, 53BP1, and PUMA expression in *PUMA* WT and KO PSCs or HPCs after IR. PSCs and HPCs were treated with 2 Gy IR, and then collected at 0 or 8 h post-IR for IB analysis. Actin was used as a control. **b** Quantification of positive cells with Rad51 foci in *PUMA* WT and KO PSCs at indicated time points after IR. **c** Representative images of Rad51 foci in *PUMA* WT and KO PSCs after IR. Scale bars, 10 μm. **d** Effects of PUMA re-expression on expression of Rad51 and 53BP1 in *PUMA* KO PSCs after IR. Lentivirus-mediated PUMA was infected into *PUMA* KO PSCs, and then selected the clones of transfected cells expressing a similar level of PUMA as that *PUMA* WT cells. **e** Effect of re-expression of PUMA on HR repair in *PUMA* KO PSCs. **f** IB of expression of Rad51 and 53BP1 in PSCs or HPCs with a Rad51 shRNA. **g** Effect of *Rad51* knockdown on PUMA-mediated HR repair. Data are representative of three independent experiments with similar results. Error bars, SD. *P < 0.05, **P < 0.01, ***P < 0.001

To elucidate the molecular mechanism by which *PUMA* deficiency promotes DSB repair, we investigated the effects of *PUMA* KO on the expression of the important proteins involved in HR and NHEJ.^16^ As shown in Fig. 2a, unirradiated *PUMA* WT and KO PSCs expressed a low level of the HR proteins Rad51, BRCA1 (breast cancer type 1), MRE11 (DSB repair nuclease), and RPA1 (replication protein A1), the NHEJ protein 53BP1, p53, and p21. Their expression was elevated after IR (Fig. 2a). However, compared with WT PSCs, *PUMA* KO PSCs expressed a significantly higher protein level of Rad51 and p21, whereas the levels of other proteins in these cells were similar between the two types of cells (Fig. 2a). Rad51 plays a central role in HR repair^17,18^, and p21 is a major target of p53 activity and has been reported to be associated with DNA repair via NHEJ.^19,20^ Thus, PUMA may regulate HR and NHEJ repair via Rad51 and p21, respectively.

Next, we measured mRNA expression for these DNA damage repair proteins and found no significant changes in their expression in *PUMA* WT and KO PSCs after IR, except *p21* mRNA, which was elevated in *PUMA* KO cells after IR (Supplementary Fig. 2a). We further determined the effects of *PUMA* KO on the mRNA expression of *ATR* (serine/threonine kinase), *ATM* (ataxia telangiectasia mutated), and another NHEJ gene *XRCC4* (X-ray repair cross complementing 4)^16^ and found that the expression of *XRCC4*, but not *ATM* or *ATR* was elevated in *PUMA* KO PSCs after IR (Supplementary Fig. 2a). Since XRCC4 is a key regulator for NHEJ repair,^21^ this data further supports that PUMA regulates NHEJ repair. We then performed immunofluorescence staining to quantify Rad51 and 53BP1 foci-positive cells in response to IR. Consistent with the results in Rad51 protein levels in Fig. 2a, *PUMA* KO significantly increased the formation of Rad51 foci compared with the WT in PSCs (Fig. 2b, c). Although 53BP1 mRNA expression was not induced in *PUMA* KO PSCs by IR, *PUMA* KO significantly increased the formation of 53BP1 foci in *PUMA* KO PSCs compared to that in the WT PSCs (Supplementary Fig. 2b, c), suggesting that PUMA may inhibit NHEJ repair.

Given that Rad51 is a critical regulator for HR repair and the mechanism by which PUMA regulates Rad51 protein levels is still unknown, we next investigated the mechanism by which PUMA regulates Rad51 protein expression and HR DSB repair. First, we transfected *PUMA* KO PSCs with *PUMA* and selected the clones of transfected cells expressing a similar level of PUMA as that *PUMA* WT cells. Compared to the empty vector (EV) control, re-expression of PUMA inhibited IR-induced Rad51 upregulation, but not 53BP1 expression (Fig. 2d), and suppressed *PUMA* KO-induced HR (Fig. 2e) and NHEJ (Supplementary Fig. 3a) repair to a level similar to that seen in *PUMA* WT PSCs. We also constructed a doxycycline-inducible PUMA system, and found that inducible expression of PUMA suppressed IR-induced HR and NHEJ repair in PSCs and somatic HEK293T cells (Supplementary Fig. 3b, c), suggesting that PUMA overexpression suppresses DSB repair. We then knocked down *Rad51* using a shRNA and found no significant difference in cell proliferation between *PUMA* WT and KO cells, with or without the transfection of Rad51 shRNA at 8 h post-IR (Supplementary Fig. 3d). Knockdown (KD) of *Rad51* did not impair 53BP1 protein expression (Fig. 2f), but inhibited HR repair in WT PSCs and HPSCs after IR (Fig. 2g). Moreover, *Rad51* KD attenuated *PUMA* KO-enhanced HR repair (Fig. 2g). Taken together, these results demonstrate that *PUMA* deficiency can promote IR-induced DSB repair via Rad51-mediated HR pathway in PSCs and HPSCs.

### PUMA binds to cytoplasmic Rad51 and regulates its ubiquitination and degradation

**Fig. 3:**
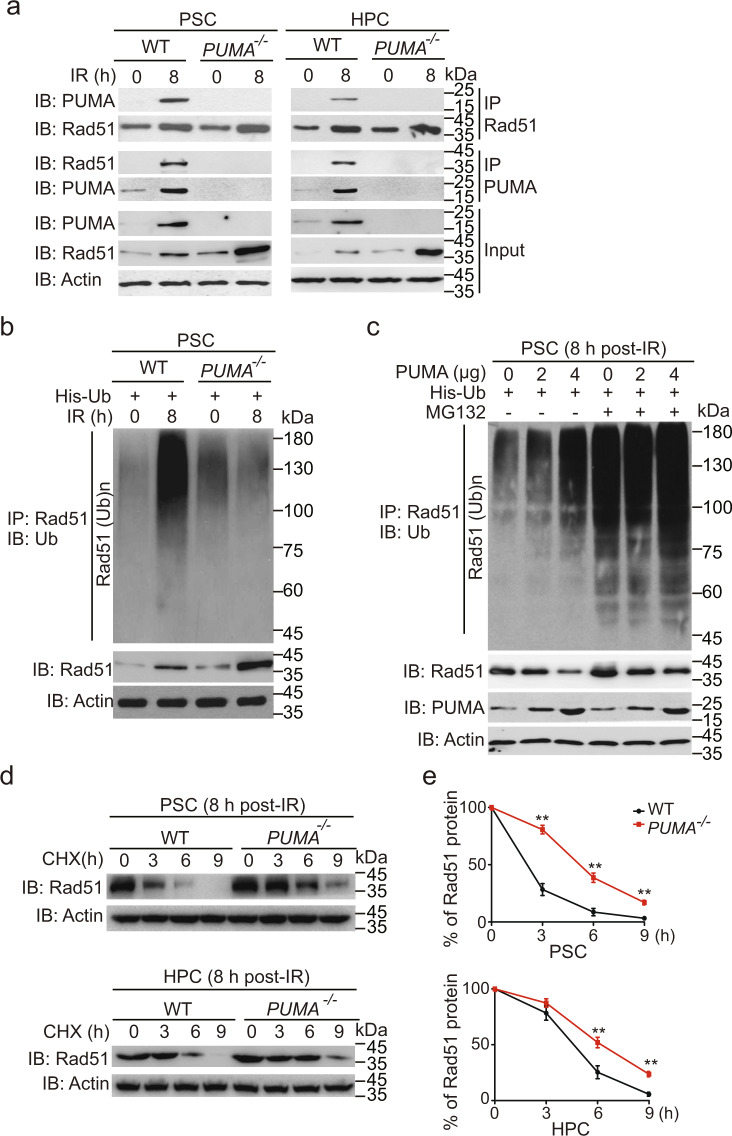
PUMA binds to cytosolic Rad51 and promotes its ubiquitination and stability. **a** Immunoprecipitation (IP) and IB of PUMA interaction with Rad51 in PSCs or HPCs. PSC and HPC cells were treated with 2 Gy of IR, and then collected at 0 or 8 h for IP and IB analyses. β-Actin was used as a loading control. **b** IP and IB of Rad51 ubiquitination in PSCs treated with or without 2 Gy IR. His-tagged Ub was transfected into *PUMA* KO PSCs, and then cells were lyzed for IP and IB analyses. **c** Effects of re-expression of PUMA in *PUMA* KO PSCs on Rad51 ubiquitination. PUMA cDNA (0, 2, or 4 μg) was transfected into *PUMA* KO PSCs. At 48 h after transfection, cells were treated with or without of 30 μM MG132 for 4 h. **d** Effects of *PUMA* knockout on Rad51 stability. At 8 h after IR, cells were treated with cycloheximide (CHX, 20 mg/ml) for the indicated time points. **e** Quantification of Rad51 protein levels in **d**. Data are representative of three independent experiments with similar results. Error bars, SD. **P < 0.01

Based on the aforementioned results that PUMA regulates the expression of Rad51 at the posttranscriptional level, we postulated that PUMA may modulate Rad51 ubiquitination and degradation. To test this possibility, we firstly determined if PUMA binds to Rad51. As shown in Fig. 3a, immunoprecipitation (IP) analyses showed that PUMA is associated with Rad51 in *PUMA* WT, but not KO PSCs and HPSCs after IR.

Next, *PUMA* WT and KO PSCs were transfected with His-tagged ubiquitin (His-Ub) and then exposed to IR or not. As shown in Fig. 3b, Rad51 was weakly ubiquitinated without IR. However, after IR, ubiquitinated Rad51 was markedly increased. Compared with the *PUMA* WT cells, KO cells showed no change in Rad51 ubiquitination at the basal level, whereas Rad51 ubiquitination in *PUMA* KO cells was significantly reduced after IR (Fig. 3b). To further confirm our finding, we transfected *PUMA* KO cells with WT *PUMA* along with different amount of His-Ub and found that the ectopic expression of PUMA in the cells promoted Rad51 ubiquitination after IR (Fig. 3c), which became more visible after the cells were treated with the proteasome inhibitor MG132 to inhibit Rad51 ubiquitination and degradation (Fig. 3c). Consistent with this, KO of *PUMA* significantly attenuated the degradation of endogenous Rad51 in PSCs and HPCs in response to IR (Fig. 3d, e). This result demonstrates that PUMA can promote Rad51 ubiquitination and degradation in PSCs and HPCs in response to IR.

### PUMA associates with cytoplasmic Rad51 and EMI1

**Fig. 4:**
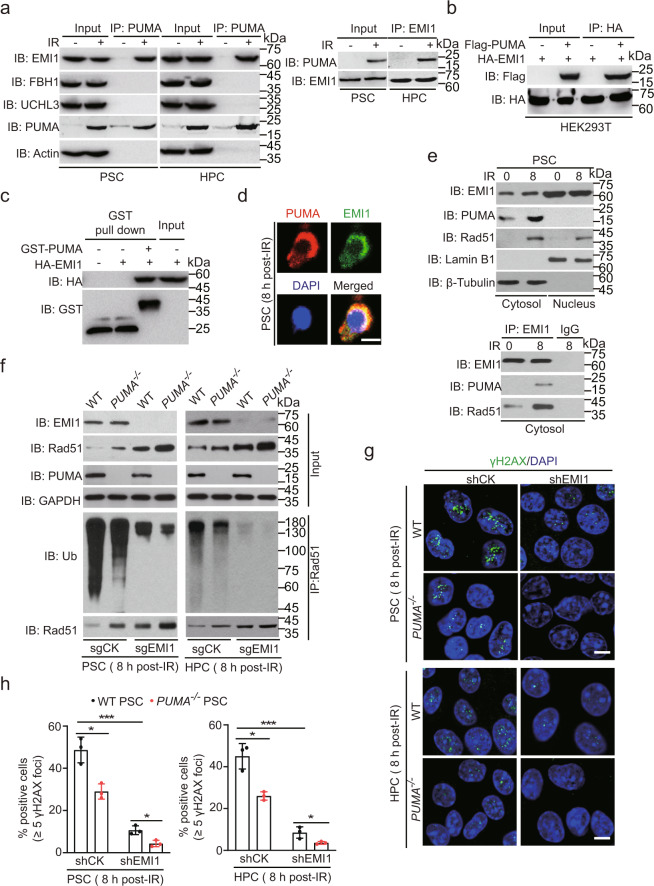
PUMA interacts with cytoplasmic Rad51 and EMI1. **a** IP and IB of PUMA interaction with EMI1, FBH1, and UCHL3 in PSCs and HPCs with or without IR. **b** IP and IB of PUMA interaction with EMI1 in HEK293T cells. **c** In vitro GST pull-down analysis. Purified GST-PUMA or GST proteins were mixed with cell extracts from PSCs. **d** Representative images of colocalization of PUMA and EMI1 in PSCs at 8 h post-IR. DAPI was used to show nucleus. An anti-EMI1 antibody (JG35-83, #NBP2-76833, Novus Biologicals) was used. Scale bars, 5 μm. **e** IP and IB of EMI1 interaction with cytosolic PUMA and Rad51 in PSCs with or without IR. **f** Effects of *EMI1* KO with a sgRNA on PUMA-mediated Rad51 ubiquitination in PSCs and HPCs after IR. **g** Representative images of γH2AX foci in *PUMA* WT and KO PSCs or HPCs transduced with or without an *EMI1* sgRNA after IR. Scale bars, 10 μm. **h** Quantification of γH2AX foci in **g**. The percentage of positive cells (≥5 γH2AX foci) was shown. Data are representative of three independent experiments with similar results. Error bars, SD. *P < 0.05, ***P < 0.001

Rad51 ubiquitination was reported to be regulated by EMI1,^12^ F-box DNA helicase 1 (FBH1, also known as FBXO18 or FBX18),^22^ and ubiquitin C-terminal hydrolase L3 (UCHL3).^23^ Thus, we performed IP and Immunoblotting (IB) analyses of PUMA, EMI1, and Rad51 in PSCs and HPCs with or without IR. As shown in Fig. 4a, endogenous PUMA interacted with EMI1, but not with FBH1 or UCHL3. Next, we co-expressed Flag-PUMA with HA-EMI1 in HEK293T cells and found that PUMA bound to EMI1 (Fig. 4b). To further validate this observation, we performed glutathione S-transferase (GST) pull-down analysis and found that purified recombinant PUMA interacted with EMI1 (Fig. 4c).

Since EMI1 was shown to be localized not only in the nucleus but also in the cytoplasm, we determined whether PUMA colocalizes with EMI1 in the cytoplasm. As shown in Fig. 4d, IF staining showed that PUMA and EMI1 colocalized in the cytoplasm in PSCs after IR. We further performed cytoplasmic and nuclear fractionation, and found that PUMA interacted with EMI1 and Rad51 in the cytoplasm in PSCs after IR (Fig. 4e). The significant level of cytoplasmic Rad51 had been observed in numerous studies,^24,25^ which contributed to a DNA damage-induced increase in nuclear Rad51 levels.^26^ Thus, these results demonstrate that PUMA interacts with cytoplasmic EMI1 and Rad51.

Given that EMI1-promoted Rad51 ubiquitination in response to the treatment with a PARP inhibitor,^12^ we determined whether EMI1 regulates PUMA-mediated Rad51 ubiquitination and DNA repair by knocking out EMI1, using a single-guide RNA (sgRNA) in *PUMA* WT and KO PSCs or HPCs. As shown in Fig. 4f, *EMI1* KO decreased Rad51 ubiquitination in WT PSCs or HPCs after IR compared with the control. Moreover, KO of *EMI1* further decreased Rad51 ubiquitination and increased Rad51 protein levels in *PUMA* KO PSC or HPC cells, suggesting that PUMA may facilitate EMI1-mediated Rad51 ubiquitination and degradation. Consistent with this observation, *EMI1* KO decreased γH2AX foci formation in WT PSCs or HPCs and further in *PUMA* KO cells (Fig. 4g, h). These data provide further support to the notion that PUMA inhibits DNA repair in a EMI1-dependent manner.

### PUMA promotes Rad51 ubiquitination by EMI1

**Fig. 5:**
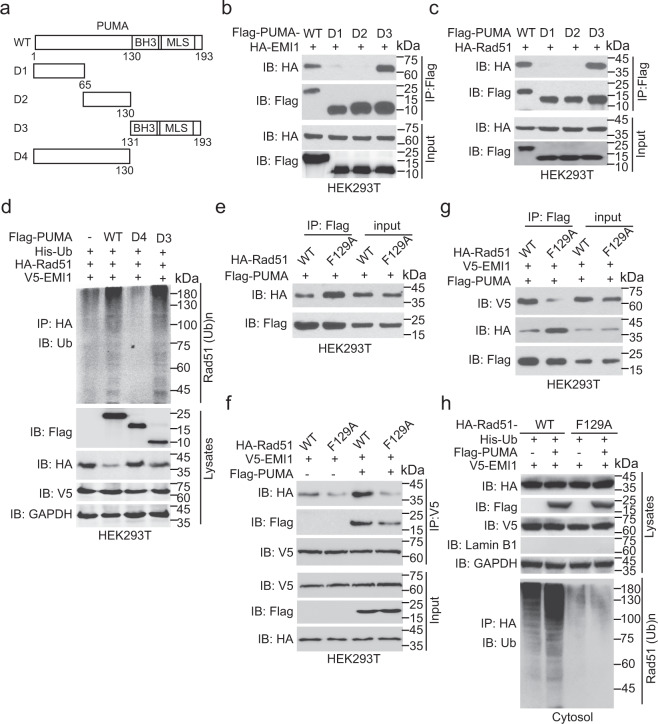
PUMA promotes Rad51 ubiquitination by EMI1. **a** Schematics of PUMA WT and truncated constructs. **b** IP and IB of the interaction of PUMA mutants with EMI1 in HEK293T cells. Flag-tagged PUMA WT or the truncation mutants with HA-EMI1 was transfected into HEK293T cells. **c** IP and IB of the association of PUMA mutants with Rad51 in HEK293T cells. **d** Effects of ectopic expression of the D3 or D4 mutant of PUMA on EMI1-mediated Rad51 ubiquitination. Flag-tagged PUMA WT, the truncation mutants, or an empty vector with His-Ub, HA-Rad51, and V5-EMI1 was transfected into HEK293T cells. **e** IP and IB of PUMA interaction with Rad51 F129A mutant. **f** PUMA increases EMI1 association with Rad51 WT, but not the F129A mutant. V5-EMI1 and HA-Rad51 WT or F129A mutant with or without PUMA were co-expressed in HEK293T cells. **g** Effect of Rad51 F129A mutation on PUMA interaction with EMI1. **h** PUMA promotes EMI1-mediated the ubiquitination of cytosolic Rad51 WT, but not the F129A mutant. Data are representative of three independent experiments with similar results. Error bars, SD. *P < 0.05, **P < 0.01

To understand how PUMA promotes Rad51 ubiquitination by EMI1, we constructed three PUMA-truncated mutants (Fig. 5a). When HA-tagged EMI1 was co-expressed with each of these PUMA-truncated mutants, D1, D2, and D3 in HEK293T cells, EMI1 interacted with the D3, but not D1 or D2, suggesting that the C-terminal fragment (amino acids 131–193) of PUMA is required for its interaction with EMI1 (Fig. 5b). We further detected Rad51 interaction with PUMA truncation mutants and found Rad51 also interacted with the D3, but not D1 or D2 (Fig. 5c). In addition, compared to the EV control, ectopic expression of WT PUMA promoted EMI1-mediated Rad51 ubiquitination (Fig. 5d). Ectopic expression of PUMA D3 mutant but not that of the D4 mutant enhanced EMI1-mediated Rad51 ubiquitination (Fig. 5d). These data demonstrate that the C-terminus of PUMA binds to both EMI1 and Rad51 to promote Rad51 ubiquitination and degradation.

To demonstrate how PUMA promotes EMI1-mediated Rad51 ubiquitination, we co-expressed Flag-tagged PUMA with HA-Rad51 WT or F129A mutant in HEK293T cells to investigate whether F129A mutation impairs PUMA association with Rad51. The F129A mutant was reported to impair Rad51 binding to EMI1.^12^ As shown in Fig. 5e, we found that compared to Rad51 WT, F129A mutation promoted Rad51 association with PUMA. We further co-expressed V5-EMI1 and HA-Rad51 WT or F129A mutant with or without PUMA in HEK293T cells, and revealed that the F129A mutation decreased Rad51 association with EMI1 and PUMA promoted EMI1 association with Rad51 WT, but not the F129A mutant (Fig. 5f). In addition, the F129A mutation attenuated EMI1 association with PUMA (Fig. 5g), suggesting that Rad51, PUMA, and EMI1 may form a ternary complex and the F129A mutation may change the structure around the F129 residue to affect its association with PUMA and EMI1. We then performed cytoplasmic and nuclear fractionation and found that F129A mutation inhibited EMI1-mediated cytosolic Rad51 ubiquitination in HEK293T cells (Fig. 5h), which is consistent with a previous report.^12^ Ectopic expression of PUMA increased EMI1-mediated cytosolic Rad51 ubiquitination and the F129A mutant impaired PUMA-enhanced Rad51 ubiquitination (Fig. 5h). Taken together, these data demonstrate that PUMA increases EMI1 association with Rad51, and thereby promotes EMI1-mediated cytosolic Rad51 ubiquitination.

### Knockout of PUMA promotes IR-induced Rad51 nucleus translocation and focus formation, HR repair, and cell survival

**Fig. 6:**
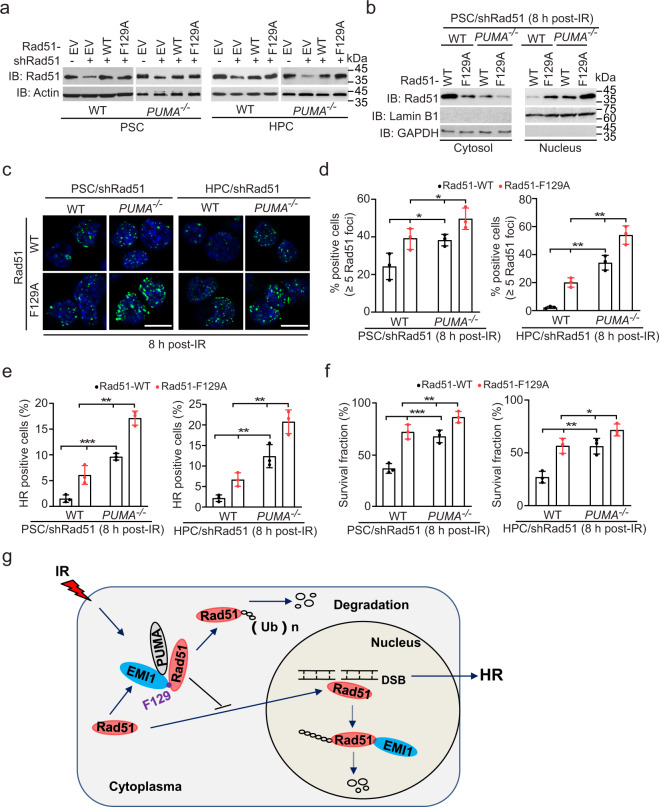
Knockout of *PUMA* promotes IR-induced cytoplasm-to-nucleus translocation and focus formation of Rad51, HR repair, and cell survival. **a** IB of re-expression of shRNA-resistant Rad51 WT or the F129A mutant in *PUMA* WT and KO PSC/shRad51 and HPC/shRad51 cells. **b** Protein expression levels of Rad51 WT and F129A mutant in the cytosol and the nucleus in PSC/shRad51 cells after IR. Lamin B1 and GAPDH are the markers for nuclear and cytosolic fraction. **c** Representative images of effects of re-expression of the F129A mutant on Rad51 focus formation in *PUMA* WT and KO PSC/shRad51 or HPC/shRad51 cells after IR. DAPI was used to show nucleus. Scale bars, 10 μm. **d** Quantification of positive cells with Rad51 foci in **c**. **e** Effects of re-expression of the F129A mutant on HR repair. **f** Effects of re-expression of the F129A mutant on cell survival after IR. Cell survival analysis was performed according to the MTT assay method with a Cell Titer 96 Aqueous Cell Proliferation Assay kit. **g** A working model. IR-induced PUMA interacts with cytoplasmic Rad51 and EMI1, which facilitates EMI1-mediated Rad51 ubiquitination and degradation, thereby leading to the inhibition of Rad51-mediated promoted DNA repair and enhancement of IR-induced cell death. Data are representative of three independent experiments with similar results. Error bars, SD. *P < 0.05, **P < 0.01

The nuclear translocation of Rad51 induced by IR or genotoxic stress is required for its role in repair by HR,^26–28^ which is consistent with the reports that Rad51 focus formation requires Rad51 translocation into the nucleus after DSB induction.^26,28,29^ To explore whether PUMA affects Rad51 nucleus translocation, we re-expressed shRNA-resistant Rad51 WT and F129A mutant in *PUMA* WT/shRad51 and *PUMA* KO/shRad51 PSCs and HPCs (Fig. 6a). Compared to the WT, *PUMA* KO decreased Rad51 WT protein accumulation in cytosol and increased its accumulation in nucleus after IR (Fig. 6b). The F129A mutation of Rad51 further decreased the levels of cytosolic Rad51 protein and increased nuclear Rad51 protein accumulation (Fig. 6b). This data suggests that *PUMA* KO may promote Rad51 cytoplasm-to-nucleus translocation probably in part by inhibiting cytosolic Rad51 ubiquitination and degradation.

Next, we determined the effects of *PUMA* KO on Rad51 focus formation, HR repair, and cell survival in response to IR. Compared with WT Rad51, re-expression of shRNA-resistant Rad51 F129A mutant increased Rad51 focus formation in *PUMA* WT PSCs and HPCs after IR (Fig. 6c, d). Re-expression of the F129A mutant in *PUMA* KO PSCs and HPCs further enhanced Rad51 focus formation (Fig. 6c, d). Then, we performed HR repair analysis and revealed that compared to Rad51 WT, re-expression of the F129A mutant markedly promoted HR repair in *PUMA* WT and KO PSCs and HPCs (Fig. 6e). Moreover, the enhancement of HR repair was the highest in *PUMA* KO cells (Fig. 6e). Consistent with *PUMA* KO-enhanced Rad51 focus formation and HR repair, compared to Rad51 WT, re-expression of the F129A mutant increased cell radioresistance in PSC/shRad51 and HPC/shRad51 cells (Fig. 6f). In addition, *PUMA* KO in combination with re-expression of the F129A mutant further enhanced stem cell radioresistance (Fig. 6f), suggesting that targeting the PUMA-EMI1-Rad51 axis has the potential to confer a survival advantage to stem cells. Taken together, our data demonstrate that IR-induced PUMA interacts to both cytosolic Rad51 and EMI1, and promotes Rad51 association with EMI1, which facilitates EMI1-mediated cytosolic Rad51 ubiquitination and degradation, thereby leading to inhibition of Rad51-mediated HR repair and enhancement of IR-induced cell death (Fig. 6g).

## Discussion

Molecular mechanisms underlying DNA repair in stem and progenitor cells are essential in assuring the quality of tissue regeneration after damage. Previous studies by our and other laboratories have shown that the loss of *PUMA* led to the long-term survival of mice following lethal irradiation,^9,10,30^ which was largely attributed to the high resistance of tissue stem and progenitor cells within the radiosensitive tissues, such as hematopoietic system and intestine to IR. Interestingly, however, tumorigenesis was not increased within those long-term survivors after lethal irradiation in the absence of PUMA. Moreover, in our recent study with induced pluripotent stem cells (iPSCs), we observed that *PUMA* deficiency was associated with reduced DNA damage and fewer chromosomal aberrations in iPSCs, as opposed to p21 or p53 deficiency.^31^ These beneficial effects of PUMA deletion cannot be explained by the previously documented role of PUMA in the apoptotic pathway. In this study, we demonstrate a novel mechanism by which *PUMA* KO enhances IR resistance in PSCs and HPCs through elevating DSB DNA repair.

As far as our knowledge, we report for the first time that PUMA negatively regulates DSB DNA repair. Our data demonstrated that the expression of Rad51 protein, a critical regulator of HR repair, is upregulated by *PUMA* KO in response to IR. PUMA binding to EMI1 and Rad51 in the cytoplasm promoted EMI1-mediated cytoplasmic Rad51 ubiquitination and degradation, and thereby inhibited Rad51 nuclear translocation and HR DNA repair, leading to increased sensitivity to IR in PSCs and HPCs. Our data are consistent with previous reports that the nuclear translocation of Rad51 induced by IR or genotoxic stress is required for its role in DNA repair by HR^26–28^ and Rad51 ubiquitination reduces Rad51 cytoplasm-to-nucleus translocation.^22,27^ Since double KO of *PUMA* and *EMI1* further increased Rad51 protein stability in PSCs and HPCs and a couple of E3 ligases were reported to regulate Rad51 ubiquitination,^22,23,27^ it is possible that other E3 ligase also collaborates with PUMA in Rad51 ubiquitination process. Our results also revealed that PUMA negatively regulates NHEJ DNA repair in PSCs and HPCs. Although the mechanism by which PUMA modulates NHEJ DNA repair is still unclear, our data suggest that it may be related to p21 and XRCC4 in the regulation of NHEJ repair.^16,19,20^ This phenomenon warrants further investigation. NHEJ activation provides an additional and plausible explanation for why *PUMA* deletion can result in improvements in the maintenance of PSC reprogramming and cell survival, without resulting in chromosomal instability.

Given our new results shown here, targeted inhibition of PUMA seems to have dual beneficial effects against IR, i.e., protecting the cells from IR-induced apoptosis and maintaining genomic stability. It was reported that PUMA can mediate IR-induced bone marrow leukocyte attrition by induction of apoptosis, which in turn stimulates the proliferation of HSPCs to repopulate the depleted compartments.^32^ Since the irradiated HPCs may carry IR-induced oncogenic lesions, their proliferation and expansion may eventually lead to malignant transformation. In contrast, in *PUMA*-deficient mice, leukocyte survival removes impetus for mutant stem cells to repopulate, thus prevents IR-induced thymic lymphoma development.^32^ *PUMA* deficiency also increases reprogramming efficiency in the absence of c-Myc, and this may be due to elevated cellular senescence to eliminate DNA damage induced by reprogramming into iPSCs.^33^ We previously demonstrated a beneficial effect of *PUMA* deletion as opposed to *p21* or *p53* absence on the chromosomal stability in established iPSC lines.^31^ Although context-dependent outcomes of *PUMA* deletion have been reported, overlapping mechanisms between DNA damage and oncogenesis exemplified by the p53 pathway represent a major challenge for the therapeutic use of stem cells.^34,35^ Given the comparable levels of PSC reprogramming, preservation of cell survival is accompanied by reduced DNA damage, fewer chromosomal alterations, as well as less strong resistance to radiation or DNA damage-induced apoptosis without increased incidences of malignancies in the absence of PUMA,^10,30,32,36^ PUMA may serve as a more desirable target for selective protection of normal tissue and stem cells than other molecules in the p53 pathway.

In summary, our findings reveal a novel, yet unconventional mechanism of PUMA in response to DNA damage and this mechanism is independent of cell apoptosis. Therefore, this new mechanistic insight further justifies that PUMA is an attractive target in stem and progenitor cells to enhance tissue regeneration after DNA damage. Although this possibility remains to be fully explored in varied preclinical settings, our current study may hold a promise for clinical applications by targeting PUMA in the patients with defective HR repair or undergoing DNA damaging regimen.

## Materials and methods

### Cell culture and ionizing radiation

Wild-type (D3 strain, ATCC) and *PUMA* KO mouse PSCs were cultured on irradiated MEF (CF1 strain, Chemicon). Wild-type and *PUMA* KO murine HPCs, IL-3-dependent cell lines, were cultured in IMDM (Gibco) with IL-3. Cells were exposed to 2 Gy IR in a Shepherd Mark I 68 Irradiator (JL Shepherd).

### Antibodies and reagents

The following antibodies were used in our studies: GST (1:1000, sc-138) and PUMA (1:10, sc-19187; Santa Cruz Biotechnology); FBXO18 (Fbh1; 1:500, #ab58881), 53BP1 (1:500, #ab175933), PUMA (1:1000 for IB, ab4963), Lamin B1 (1:1000, ab133741), UCHL3 (1:1000, ab244371), RPA70 (1:1000, ab176467), MRE11 (1:500, ab214), BRCA1 (1:1000, ab131360), and Rad51 (1:10, 51RD01; Abcam); anti-γH2AX [phosphor-Histone H2A.X(Ser139) (1:1000, 20E3, Cell Signaling Technology); anti-Flag M2 (1:1000 for IB, 1:100 for IF, F3165, Sigma-Aldrich); anti-HA (1:2000 for IB, 1:100 for IF, #66006-1-Ig), anti-β-actin (1:5000, #66009-1-Ig), and anti-lamin B1(1:5000, #66095-1-Ig; Proteintech Group); and anti-EMI1 antibody (1:50 for IF, JG35-83, #NBP2-76833, Novus Biologicals or 1:1000 for IB, #385000, Life Technologies). The secondary antibodies were from Life Technology or Jackson ImmunoResearch Laboratories. Cell culture media and other reagents were from Hyclone, Invitrogen, Sigma-Aldrich, and Fisher Scientific.

### Immunofluorence staining assay

PSCs were grown on coverslips for 12 h before IR. For HPCs, irradiated cells were cytospun onto each slide using CytoSpin (Thermo Fisher Scientific). Cells were fixed, permeabilized, blocked, and stained with each antibody. For nuclear foci observation, nucleus images were acquired using a Zeiss Axio Observer.Z1 microscope and AxioVision (4.7.1.0) software (Carl Zeiss Microimaging Inc.). For subcellular localization observation, each slide was photographed using a FluoView FV1000 confocal microscope and FV10-ASW (02.01.01.04) software (Olympus).

### Comet assay

Comet assay was performed as we previously described.^37^ Cells were harvested at various times post-IR and processed for alkaline comet assay using a Comet assay^®^ kit (Trevigen), according to the manufacturer’s protocol. Each slide was photographed under a Zeiss Axio Observer Z1 microscope, and the percentage of tail intensity was computed by the Comet Assay IV software (Perceptive Instruments Ltd.). For each analysis, 200 cells were processed, and each experiment was repeated three times.

### HR and NHEJ assays

The HR and NHEJ assays were performed as we previously described.^37^ Briefly, we generated *PUMA* WT or KO PSCs and HPCs with stable expression of a pDR-GFP or pEJ5-GFP reporter^38^ for HR or NHEJ, respectively. I-SceI expression vector (pCBA-I-SceI) was transfected into the cells. The parallel transfection with pDsRed2-ER (Clontech) was used to normalize for transfection efficiency. At 48 h after I-SceI transfection, cells were pretreated with 2 Gy IR and then harvested at 8 h post-IR for analysis, using flow cytometry to examine recombination induced by I-*Sce*I digestion. Dead cells were excluded by PI staining. For each analysis, 20,000 cells were processed, and each experiment was repeated three times.

### Quantitative real-time RT-PCR assay

Total RNAs were isolated from each cell using a RNeasy Mini Kit (Qiagen) and reverse-transcribed using ImProm-II^TM^ Reverse Transcriptase (Promega) according to the manufacturer’s protocol. PCR was performed using DyNAmo HS SYBR Green qRT-PCR kit (Finzymes) and a 7500 Fast Real-Time PCR System (Applied Biosystems). The relative quantitative value of the Rad51 was normalized against β-actin. The primers were listed in Supplementary Table 1.

### Plasmids

PUMA, Rad51, and EMI1 cDNAs were gained from DNA core in Shanghai Jiao Tong University and then were sub-cloned into a pcDNA3 or pLVX vector.

### Single-guide RNA knockout, shRNA knockdown, and transfection assays

Sequences of sgRNAs were designed using the MIT online tool (http://crispr.mit.edu). shRNAs were purchased from GeneChem (Shanghai, China). The plasmid transfections and shRNA infections were carried out, as we previously described.^39^ Briefly, HEK293T cells were transfected with specified DNA and packaging plasmids. Then, viruses were collected, concentrated, and transduced into various cells, as we previously described.^39^

### Immunoblotting and immunoprecipitation and ubiquitination assays

IB and IP and ubiquitination assays were performed, as we previously described.^39^ Briefly, cells were lysed in 20 mM Tris-HCl, pH 7.5, 150 mM NaCl, 1 mM EDTA, 2 mM Na3VO4, 5 mM NaF, and 1% Triton X-100 buffer with Protease inhibitor cocktail (Thermo). For IP and ubiquitination assays, the protein lysates were incubated with appropriate antibodies, captured by protein A/G plus-agarose (Santa Cruz), and eluted by sample buffer. The proteins were resolved in SDS–polyacrylamide gel electrophoresis, transferred to polyvinylidenedifluoride membrane, and detected by the appropriate antibodies.

### Purification of recombinant proteins and GST pull-down assay

Purification of recombinant proteins was performed, as we previously described.^39^ Briefly, GST-PUMA in the plasmid pGEX-4T-1 was transformed into *Escherichia coli* BL21 and purified using glutathione beads, according to the manufacturer’s procedures. Pull-down analysis was performed by incubating purified GST-PUMA protein with the cell extracts from PSCs.

### Cell apoptosis analysis

Cell apoptosis analysis was performed using a Annexin V-FITC Apoptosis Detection Kit (R&D Systems, Inc., Minneapolis, MN) according to the manufacturer’s recommendation. The flow cytometry data were analyzed by Syan software as we previously described.^31^

### Cell survival analysis

Cell survival analysis was performed according to the MTT assay method with a Cell Titer 96 Aqueous Cell Proliferation Assay kit. Briefly, cells were collected at day 3 after 2 Gy IR, and 10 μl of 4 mg/ml MTT solution was added to each well of the 96-well plate. The cells were subsequently incubated for 4 h in the dark. The absorbance was measured in a microplate reader at 490 nm, and the results were expressed as a percentage of the control.

### Statistical analysis

The data were analyzed using GraphPad Prism version 5.0 for Windows (GraphPad Software Inc.). *P* values were calculated using an unpaired two-tailed Student’s *t* test. *P* values < 0.05 were considered significant.

## Supplementary information

Supplemental material

## Data Availability

The data supporting the finding of this study are available within the article and its Supplementary Information files or available from the corresponding author on reasonable request.
