# Supplementary material for: PUMA facilitates EMI1-promoted cytoplasmic Rad51 ubiquitination and inhibits DNA repair in stem and progenitor cells
Source: Signal Transduct Target Ther. 2021 Mar 31;6:129. doi: 10.1038/s41392-021-00510-w (PMC8009889; doi:10.1038/s41392-021-00510-w)
Supplement: Supplementary file 1 — Supplemental material [file 41392_2021_510_MOESM1_ESM.docx]

Supplementary Materials for

PUMA facilitates EMI1-promoted cytoplasmic Rad51 ubiquitination and inhibits DNA repair in stem and progenitor cells

Jin Wook Kang, Zhiyan Zhan, Guangzhen Ji, Youzhou Sang, Daohong Zhou, Yanxin Li, Haizhong Feng, and Tao Cheng

Correspondence to: [chengtao@ihcams.ac.cn](mailto:chengtao@ihcams.ac.cn)

**This PDF file includes:**

Figures. S1 to S3

Tables S1

**Figure. S1.**

**Flow cytometry analyses of γH2AX positive cells and cell apoptosis in WT and *PUMA^-/-^* PSC at indicated time point after treated with 2 Gy IR.**

(**a**) Representative images of flow cytometry analysis of γH2AX positive cells. WT and *PUMA^-/-^* PSCs were pre-treated with 2 Gy IR and then cells were collected for γH2AX staining. (**b**) Quantification of γH2AX positive cells in **a**. (**c**) Representative images of flow cytometry analysis of cell apoptosis. (**d**) Quantification of cell apoptosis in **c**. Data are representative of three independent experiments with similar results. Error bars, SD. **P* < 0.05, ***P* < 0.01.

­

**Figure. S2.**

**qRT-PCR analysis of expression of ATM, ATR, p53, p21, HR and NHEJ targets and 53BP1 focus analysis in WT and *PUMA^-/-^* PSCs with or without IR.**

(**a**) qRT-PCR analysis of expression of ATM, ATR, p53, p21, HR and NHEJ targets in WT and *PUMA^-/-^* PSCs at 0 or 8 h after 2 Gy of IR. (**b**) 53BP1 focus analysis in WT and *PUMA^-/-^* PSCs with or without IR. (**c**) Quantification of 53BP1 focus analysis. Data are representative of three independent experiments with similar results. Error bars, SD. **P* < 0.05, ***P* < 0.01.

**Figure. S3.**

**Effects of re-expression or overexpression of PUMA on NHEJ and HR repair in PSC and 293T cells and effect of Rad51 knockdown on cell proliferation after IR.**

(**a**) Corresponding to Fig. 2d, re-expression of PUMA in *PUMA* KO PSCs inhibits *PUMA* KO-upregulated Rad51 expression in response to IR. (**b**) IB of doxycycline (Dox)-inducible expression of PUMA in PSC and 293T cells. PSC and 293T cells transfected with Dox-inducible PUMA were treated with or without 2 mg/ml Dox for 6 h. (**c**) Effects of overexpression of PUMA on NHEJ and HR repair in PSC and 293T cells after IR. PSC and 293T cells transfected with Dox-inducible PUMA were pre-treated with or without 2 mg/ml Dox for 4 h and then treated with 2 Gy IR. (**d**) Effect of Rad51 knockdown on cell proliferation after IR. A Rad51 shRNA was transfected into WT and *PUMA^-/-^* PSCs. Cells were treated with 2 Gy IR and then cell proliferation was analyzed. Data are representative of three independent experiments with similar results. Error bars, SD. **P* < 0.05, ***P* < 0.01.

**Table. S1.**

**Primers used in the qRT-PCR analysis.**

| Genes | Primer Sequence (5’-3’) | |
| --- | --- | --- |
|  | Forward Reverse | |
| *Rad51* | GGTTAGAGCAGTGTGGCATAA | TAGTTCCTTCTTCGGTGCATAAG |
| *53BP1* | TGACCACTAACTCGGGTTCTA | TCTTTGCTGGGCTGTTCTATC |
| *Brca1* | CTTCTATCAGGTGTGCTCTTCC | TCTTCACTGCTACCACAACTATC |
| *Mre11* | ATTCCAGGGCTGATCAAAGG | GGTCATCGTCATCATCCTCATC |
| *Xrcc4* | TTACTGCCTGGACACCATTAC | CTTTGGCACTCACACATTTCTC |
| *Rpa1* | GTGGATGAAAGCGGTGAAATC | GGCGCCCTTTGAGAAGTAATA |
| *Tp53* | GCCATGGCCATCTACAAGAA | AATTTCCTTCCACCCGGATAAG |
| *p21* | AAGTGTGCCGTTGTCTCTTC | AGTCAAAGTTCCACCGTTCTC |
| *Atm* | GATACCAGATCCGTGGAGATTT | CTTCCCAGCCTACGTCTATTT |
| *Atr* | AACATTCGTGGCATTGACTG | AAGCAAGGTGATCTCATCCG |
| *Actb* | GAGGTATCCTGACCCTGAAGTA | CACACGCAGCTCATTGTAGA |
